# The Roles of BLH Transcription Factors in Plant Development and Environmental Response

**DOI:** 10.3390/ijms23073731

**Published:** 2022-03-29

**Authors:** Xiaolin Niu, Daqi Fu

**Affiliations:** The College of Food Science and Nutritional Engineering, China Agricultural University, Beijing 100083, China; niuxl@cau.edu.cn

**Keywords:** BLH/BELL, transcription factors, plant development, environmental stress

## Abstract

Despite recent advancements in plant molecular biology and biotechnology, providing enough, and safe, food for an increasing world population remains a challenge. The research into plant development and environmental adaptability has attracted more and more attention from various countries. The transcription of some genes, regulated by transcript factors (TFs), and their response to biological and abiotic stresses, are activated or inhibited during plant development; examples include, rooting, flowering, fruit ripening, drought, flooding, high temperature, pathogen infection, etc. Therefore, the screening and characterization of transcription factors have increasingly become a hot topic in the field of plant research. BLH/BELL (BEL1-like homeodomain) transcription factors belong to a subfamily of the TALE (three-amino-acid-loop-extension) superfamily and its members are involved in the regulation of many vital biological processes, during plant development and environmental response. This review focuses on the advances in our understanding of the function of BLH/BELL TFs in different plants and their involvement in the development of meristems, flower, fruit, plant morphogenesis, plant cell wall structure, the response to the environment, including light and plant resistance to stress, biosynthesis and signaling of ABA (Abscisic acid), IAA (Indoleacetic acid), GA (Gibberellic Acid) and JA (Jasmonic Acid). We discuss the theoretical basis and potential regulatory models for BLH/BELL TFs’ action and provide a comprehensive view of their multiple roles in modulating different aspects of plant development and response to environmental stress and phytohormones. We also present the value of BLHs in the molecular breeding of improved crop varieties and the future research direction of the BLH gene family.

## 1. Introduction

With the growth of the global population, humans are facing the challenges of increasing crop production and quality, while reducing land cultivation and responding to droughts, floods, pests and diseases due to global climate change [1]. Creating new plant varieties through biotechnology, to increase crop yields per unit area and improve their quality, is an effective way to solve the above challenges. It requires detailed knowledge of the underlying molecular mechanisms of plant development, senescence and stress responses and the identification of valuable genes and understanding of their actions, so they can be used to create new varieties through genetic engineering [2].

The specific expression of plant genes throughout the life cycle is transcriptionally regulated by transcription factors (TFs). There have been many reviews on the roles of important plant TF families, including NACs (NAM, ATAF and CUC) [3], MADS (MADS-box) [4], ERF (Ethylene Responsive Factor) [5] and TALE (Three-Amino-acid-Loop-Extension) [6]. The TALE TFs include two subfamilies, KNOX (Knotted-like Homeobox) and BLH (BEL1-like Homeobox), which play an important role in plant development and stress response [7,8,9,10]. With the rapid development of new generation sequencing technology, bioinformatics technology and molecular biology, *BLH* genes in different species have been cloned and verified. These play important roles in the regulation of plant development, hormone response and stress response [6,7,11,12,13]. Although several reviews of the TALE family have been published, they mainly focus on KNOX and there is a lack of review on the BLH subfamily [6]. A comprehensive review of BLH TFs in different species can contribute to a better understanding of the function of the BLH regulatory network and its role in plant development and stress response. Here, we review the current advances of the BLH family in different species and also explore their potential value in the molecular breeding of crops and their future research direction.

## 2. The BLH Family

**Figure 1:**
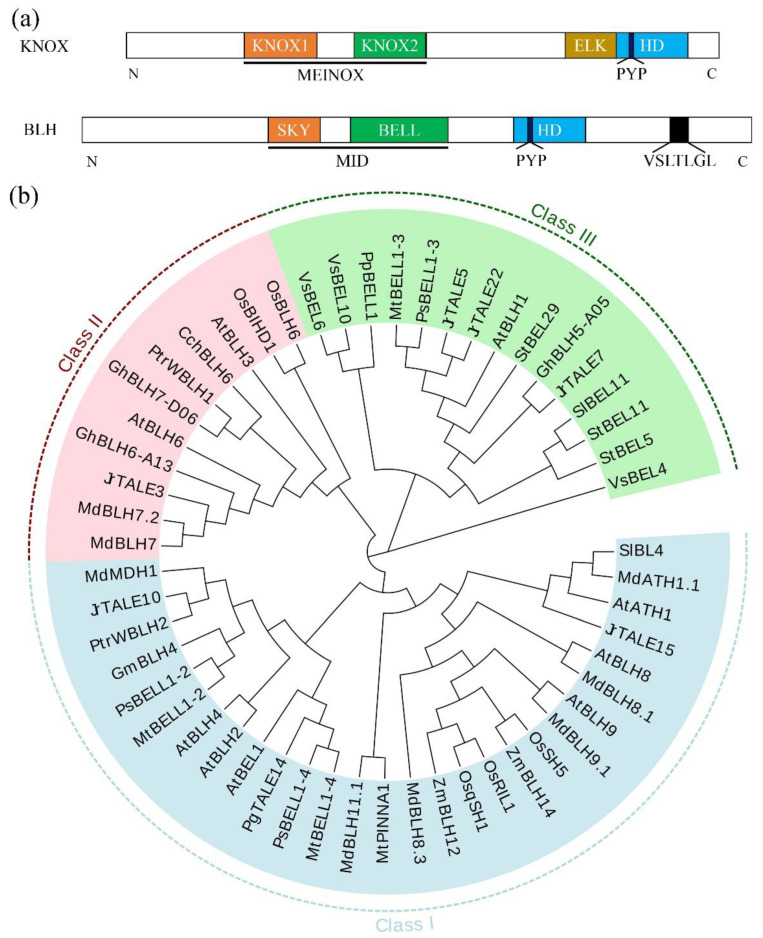
(**a**) Schematic diagram of the structure of KNOX and BLH proteins. KNOX proteins contain an ELK domain, an HD domain and an MEINOX domain which is composed of KNOX1 and KNOX2. BLH proteins contain an HD domain, a VSLTLGL domain and an MID domain which is composed of SKY and BELL. (**b**) Phylogenetic tree of 54 BLH homologous proteins that have been functionally identified. A total of 54 BLH protein sequences were aligned with ClustalX 2.1 program, and the phylogenetic tree was constructed by Neighbor-Joining method. All BLHs are divided into three class and each class is represented by different colors. Class I subfamily is represented by blue, class II subfamily is represented by pink and class III subfamily is represented by green. Gene prefixes represent different species. (Pp: *Physcomitrium patens;* Vs: *Vandenboschia speciose*; At: *Arabidopsis thaliana*; Sl: *Solanum lycopersicum*; St: *Solanum tuberosum*; Os: *Oryza sativa* L.; Zm: *Zea mays* L.; Gh: *Gossypium hirsutum* L.; Gm: *Glycine max* L.; Md: *Malus domestica* L.; Mt: *Medicago truncatula*; Ps: *Pisum sativum* L.; Cch: *Camellia chekiangoleosa*; Ptr: *Populus trichocarpa*; Jr: *Juglans regia* L.; Pg: *Punica granatum* L.).

BLH and KNOX are characterized by three-amino-acid-loop-extensions and the conserved “PYP” (proline-tyrosine-proline) amino acid sequence in the homeodomain. However, they differ in their N-terminal and C-terminal regions (Figure 1a). KNOX proteins have a unique MEINOX domain, comprising KNOX 1 and 2 domains, and an ELK domain, while BLH proteins have an N-terminal conserved SKY domain and a BELL domain, upstream of the HD domain [6,14]. In addition, some BLH proteins contain a conserved C-terminal “VSLTLGL” sequence with unknown functions [15,16]. The SKY and BELL domains together make up the MID domain (also known as POX domain) (Figure 1a), which can interact with the MEINOX domain to form a homodimer or a heterodimer to regulate the growth and development of plants and response to stress [6,17,18]. HD domain is the DNA-binding domain [19]. In recent years, the BLH family has been identified in many species, and their functions have been deeply studied. The largest number of BLH family members is in *Gossypium hirsutum*, whereas only two members are found in *Selaginella moellendorffii* (Table 1). We also analyzed the kinship of *BLH* functional genes in different species. Phylogenetic tree analysis showed that these identified *BLHs* could be divided into three groups (class I, class II and class III) (Figure 1b). It can be found that *BLHs* involved in plant stress response are mainly concentrated in class I, while *BLHs* involved in plant development and hormone response are mainly concentrated in class II and class III (Table 1; Figure 1b).

## 3. The Function of BLHs in Regulating Plant Development

### 3.1. BLHs Regulate the Development of Plant Meristems

The apical meristem of higher plants produces different new tissues, such as roots, shoots, leaves, flowers and fruits [67]. The maintenance of meristem activity depends on the dynamic balance between cell division and cell differentiation, and BLH TFs play an important regulatory role in the development of meristems [68]. In *Arabidopsis thaliana*, *ATH1* (*ARABIDOPSIS THALIANA HOMEOBOX GENE1*), *BLH3* (*BEL1-LIKE HOMEODOMAIN GENE3*), *PNF* (*POUND-FOOLISH*) and *PNY* (*PENNYWISE*), as well as a series of *PNY* alleles *VAN* (*VAAMANA*), *BLR* (*BELLRINGER*), *LSN* (*LARSON*), *BLH9* (*BEL1-LIKE HOMEODOMAIN GENE9*) and *RPL* (*REPLUMLESS*) encode the BLH proteins and were involved in the formation and maintenance of meristems [27,30,69,70,71]. *ATH1* is expressed throughout the apical meristem and leaf primordia [72]. *BLH3* is expressed in the central region of the apical meristem [13], and *PNY* is expressed in the central region of the apical meristem and inflorescence meristem [71]. BLR can inhibit the expression of *PME5* (*pectin methyl-esterase 5*) in meristems to limit the demethylation of pectin in inflorescence meristems [73,74]. ATH1, PNY and PNF can interact with KNOX STM (SHOOT MERISTEMLESS) to form heterodimer and regulate the development of meristem [29,75,76].

*VPB1* (*verticillate primary branch 1*) belonging to *BLH* family is expressed in the shoot tip meristem at the early stage of panicle development in rice. It not only maintains the activity of the inflorescence meristem, but also plays an important regulatory role in the development of primary branch meristem structures [28]. *verticillate rachis* (*ri*) and *RI-LIKE1* (*RIL1*) are also rice *BLH* genes, which are homologous with *PNY* and *PNF,* respectively. They are expressed in restricted regions of the inflorescence meristem (IM) and primary branch meristem (PBM). The *ri ril1* double mutant cannot normally establish and maintain the shoot apical meristem (SAM) during embryogenesis [11]. In maize, the double mutants of *blh12 blh14* showed axillary meristem growth defects and could not produce normal tillers. On the other hand, Tsuda et al. (2017) found that BLH12/14 interacted with KNOTTED1 (KN1) and accumulated in the putative internode meristem of maize, while KN1 did not accumulate in the double mutant of *blh12 blh14*, suggesting that BLH transcription factors may be involved in the maintenance of meristems [51].

### 3.2. BLHs’ Regulation of Flower Development

The transition from vegetative growth to reproductive growth is a complex process, involving the regulation of many different genes and some TFs have been reported to regulate the expression of key flowering genes to ensure normal flowering at an appropriate time [77,78]. It was found that BLH TFs can regulate flowering-related genes expression in flowering plants [6].

**Figure 2:**
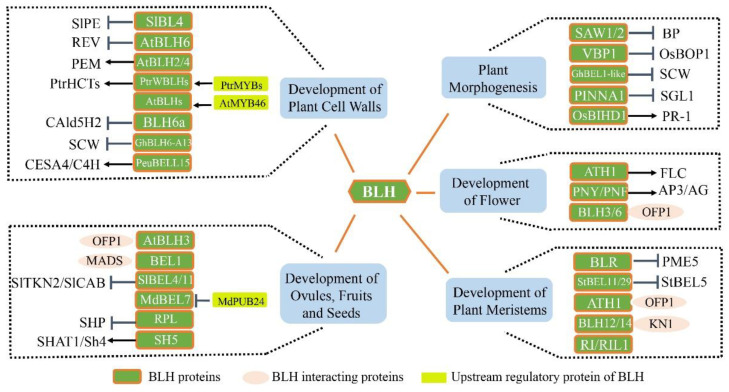
Role of BLHs in plant development in different species. The composition of BLH in signaling pathways in plant development is divided into five aspects: development of plant meristems, development of flower, plant morphogenesis, development of plant cell walls and development of ovules, fruits and seeds. The main downstream genes are depicted in the diagram. Arrows indicate promotion and vertical lines indicate repression. Gene prefixes represent different species (At: *Arabidopsis thaliana*; Sl: *Solanum lycopersicum*; St: *Solanum tuberosum*; Gh: *Gossypium hirsutum*; Mt: *Medicago truncatula*; Ptr: *Populus trichocarpa*; Os: *Oryza sativa*; Gm: *Glycine max L*; Zm: *Zea mays*; Peu: *Populus euphratica*).

The Arabidopsis, *BLH* family genes *BLH3*, *BLH6*, *BLH8* (*PNF*), *BLH9* (*PNY*/*RPL*/*BLR*) and *ATH1* play an important role in flower development and are necessary for inflorescence formation [35,37,79,80,81]. Overexpression of *BLH3* and *BLH9* lead to early flowering, while overexpression of *ATH1* and *BLH6* lead to delayed flowering [29,32,33,34]. *ATH1* is expressed in flower organs and its encoded protein inhibits flowering by regulating the expression of *FLC* (*FLOWERING LOCUS C*) [13,82]. ATH1, BLH3 and BLH6 can interact with OFP1 (OVATE FAMILY PROTEIN1) to regulate the inflorescence structure and flowering of plants [13,34,72]. The *pny pnf* double mutants can normally receive flowering induction signals, but show abnormal flowering phenotype [37,83]. *AG* (*AGAMOUS*) is the key regulator in the development of floral organs and meristems. BLR is necessary to prevent the ectopic expression of *AG* in floral meristem and can also directly inhibit the expression of *AG*. Bao et al. showed that BLR may recruit the corepressors of TFs into *AG* chromatin such as LEUNIG (LUG) and SEUSS (SEU), resulting in inhibiting the expression of *AG* and coordinating the development of floral organs [71]. In addition, PNY and PNF can interact with STM and participate in the recognition and formation of petals, stamens and pistil organs by regulating the expression of *AP3* (*APETALA3*) and *AG* [39,71,84] (Figure 2). Previous studies has found that *ATH1*, *PNY* and *PNF* played both overlapping and opposing roles in inflorescence development and the *ath1 pny pnf* triple mutant showed normal flowering but abnormal flower organ development [29]. Recent studies have found that *RPL*, which is an allele of *PNY*, is very important in the normal development of the inflorescence in *Brassicaceae*. RPL can combine with specific TFs to jointly regulate the transcription of genes related to inflorescence development and promote the normal development of inflorescence structure [85]. The *PgTALE8* from pomegranate is a homolog of *ATH1*, and recent studies have shown that it plays an important regulatory role in inflorescence development [66]. Guo et al. identified the walnut *TALE* family members and found that *BLH* genes (*JrTALE3*, *JrTALE5*, *JrTALE7*, *JrTALE10*, *JrTALE15* and *JrTALE22*) were differentially expressed at different stages of flower bud development, which provided a basis for better understanding the transformation mechanism of the walnut flower bud [65]. TaqSH1 is a transcription factor of wheat BLH family, which is homologous with RPL in Arabidopsis. The overexpression of *TaqSH1* in Arabidopsis affected the floral organ abscission in transgenic plants, and down-regulated the expression level of genes related to floral organ abscission [42].

### 3.3. BLHs’ Regulation of Plant Morphogenesis

In the stage of plant morphogenesis, new structures are formed from organ primordia, and the polar development pattern is gradually formed. In this process, the gene regulatory network is involved in the differentiation and expansion of cells, regulates the balance between morphogenesis and differentiation, and ultimately affects the size, shape and complexity of plant organs [86]. BLH TFs play an important role in the complex regulatory network of plant morphogenesis.

In Arabidopsis, the *BLH* family genes *SAW1* (*BLH2*), *SAW2* (*BLH4*), *BLH6*, *PNY* and *VAN* allele of *PNY* are involved in plant morphogenesis and play important regulatory roles [26,28,36]. The *SAW1* and *SAW2* genes are highly homologous, and overexpression of *SAW1* results in smaller overall plant morphology and loss of leaf polarity. The *saw1 saw2* double mutant results in sawtooth and revolute leaf margins, indicating these two genes act redundantly to affect leaf margin growth [36]. At the same time, both SAW1 and SAW2 can negatively regulate the expression of the *KNOX* family gene *BP* (*BREVIPEDICELLUS*) to regulate leaf edge morphogenesis [36,87]. The *pny bp* double mutant showed abnormal internode development and increased branching [28,88]. The *van* mutant showed a dwarf phenotype [27]. The *blh6* mutant inhibited the effect of *BP* on the root phenotype. Genetic analysis of hybrid lines between *BP* overexpression and *blh6* mutants proved that *BLH6* was involved in the effect of *BP* on plant configuration [26]. Ectopic expression of apple *BEL1-like* gene *MdMDH1* in Arabidopsis resulted in the dwarfing of transgenic plants and deformity of carpels and pods [58].

Rice *BLH* genes *RI* and *RIL1* play an important role in the construction of the structure of rice inflorescence, where they regulate the structure of main inflorescence branches, the secondary branches and spikelets in rice. The inflorescence of *ri ril1* double mutant showed an abnormal phenotype of increased branching [11]. *VPB1* and *OsBOP1* (*BLADE-ON-PETIOLE1*) are important regulators of inflorescence configuration [89]. VPB1 can negatively regulate the expression of *OsBOP1* and affect the development of rice panicle configuration [28]. Overexpression of a rice *BLH* gene, *OsBIHD1,* in tobacco resulted in some abnormal morphological phenotypes in top buds and roots [46]. Overexpression of barley *JuBEL2* in tobacco resulted in ectopic growth of transgenic plants. Some plants grow more branches and show abnormal bifurcation of leaf veins. When *JuBEL2* was expressed in Arabidopsis, the transgenic plants formed more shoots compared with the wild type [90]. The *blh12 blh14* double mutants of maize showed narrower leaves and failed to produce tillers or female inflorescences [51].

Overexpression of soybean *GmBLH4 (BEL1-LIKE homeodomain 4)* in Arabidopsis changed leaf phenotype and pod length [56]. The mutation of *PINNA1 (PINNATE-LIKE PENTAFOLIATA1)* of *Medicago truncatula*, a *BLH* family member, produced more compound leaves, which changed from a three leaf palm shape to a five leaf pinnate compound leaf shape [61].

### 3.4. BLHs’ Regulation of Plant Cell Wall Structure and Composition

Plant cell walls are mainly composed of cellulose, hemicellulose, pectin and lignin, and provide plants with important structural support and protection. The formation of the plant cell wall is a highly complex process in plant growth and development [91]. The synthesis of these components are regulated by a series of TFs in plants [92].

In Arabidopsis, *BLH2*, *BLH4*, *BLH6* and *BLR* are involved in the formation and development of cell walls. Studies have proved that *BLH2* and *BLH4* are significantly expressed in seed mucus-secreting cells and affect the formation of the seed cell wall (mucus). In *blh2 blh4* double mutants, the adhesion of mucus to seeds was significantly reduced [93]. In addition, BLH2 and BLH4 play a positive role in regulating the demethylation of *HG* (*homogalacturonan*) in seed mucus [31]. BLR participates in the formation of the cell wall by regulating the expression of *PME5*, a gene related to cell wall formation [94]. The homeodomain-leucine zipper (HD-ZIP) transcription factor REV (REVOLUTA) has been shown to be necessary for inter bundle fiber development [95]. BP and REV play a common role in the process of lignification, and the number of xylem cells in the secondary wall decreased in *bp rev* double mutant [94]. BLH6 negatively regulates the expression of secondary cell-wall-related genes by transcription inhibition. The secondary cell wall of the *blh6* mutant was thicker than that of wild type. BLH6 also interacts with AtKNAT7 to participate in the formation of the secondary wall by inhibiting the expression of *REV* [32,96]. GhBLH6-A13 in cotton can also regulate the development of the secondary cell wall. Heterologous overexpression of *GhBLH6-A13* in Arabidopsis significantly inhibited the synthesis of lignocellulose in the inter bundle fibers [52]. *OsBLH6* is involved in the synthesis of the secondary cell wall and its overexpression enhanced the development of the stem secondary wall, while the lignin content was decreased in *OsBLH6* knockout lines [49].

Lignin is one of the main components of the cell wall. Snapyl alcohol is an important precursor for lignin synthesis, and *CAld5H2* (*coniferaldehyde 5-hydroxylase*) is the key enzyme catalyzing sinapyl alcohol biosynthesis [97]. BLH6a in poplar is a negative regulator of *CAld5H2* and plays an important regulatory role in the synthesis of sinapyl alcohol [98]. PtrMYB021 and PtrMYB074 are transcriptional activators in the fiber of stem-differentiating xylem in *Populus trichocarpa*. *PtrWBLH1* and *PtrWBLH2* are the direct targets of PtrMYB021 and PtrMYB074, which can directly regulate 15 single molecule cellulose and cell wall cellulose biosynthesis genes. Similarly, AtMYB46 in Arabidopsis can directly regulate 17 transcription factors and 12 cell wall components, including *AtBLH2*, *AtBLH3*, *AtBLH6* and *AtBLH10* [64]. *PeuBELL15* regulates the accumulation of glucan and lignin and promotes the expression of genes related to secondary vascular growth, cellulose synthase and lignin biosynthesis in *Populus euphratica*, such as *CESA4 (Cellulose synthase 4)*, *C4H* (*cinnamate-4-hydroxylase*) and *4CL* (*4-coumarate: CoA ligase*) [99]. CchBLH6 is a positive transcriptional regulator of lignin biosynthesis during the lignification of camellia fruit in *Camellia chekiangoleosa* [63] (Figure 2). In tomato, SlBL4 can directly repress the expression of *SlPE* (*pectinesterase*), resulting in reduced texture and cell wall thinning in tomato fruits [41].

### 3.5. BLHs’ Regulation of Fruit Development

There exist complex transcript regulatory networks during fruit development. BLH TFs are one of the important regulatory factors.

*BEL1* is expressed and plays a regulatory role during ovules development of Arabidopsis. The *bel1* mutant showed ovule with a defective embryo sac. On the other hand, BEL1 interact with MADS box protein complex to control the formation of the ovule integument [100,101,102,103]. The *BLH* gene *PgTALE14* in pomegranate is also an important regulator of ovule development [66]. The *RPL* gene has been proved to be involved in the development of placental frame (replum), and RPL negatively regulates the expression of *SHP* to prevent the repeated use of the fate of valve marginal cells [30]. In rice, the *BLH* genes *qSH1* and *SH5* are involved in rice grain shedding by promoting the development of abscission zone and inhibiting lignin synthesis [47,50]. *SHAT1* (*shattering abortion 1*) and *SH4* (*shattering 4*) are the necessary regulatory factors for the formation of the abscission zone (AZ). Yoon et al. found that SH5 can induce the expression of *SHAT1* and *SH4* to promote the formation of abscission zone and seed dropping [50]. Overexpressing the barley *JuBEL1* in tobacco resulted in the phenomenon of reduced male fertility [90]. Apple MdBEL7 was degraded by ubiquitination, with the U-box E3 ubiquitin ligase MdPUB24, resulting in the degradation of chlorophyll in apple during storage [57]. In tomato, *SIBEL11* and *SlBL4* have been shown to be involved in regulating the development of chloroplasts and chlorophyll synthesis in tomato fruit [40,41] (Figure 2). Plant developmental processes involving BLH proteins are summarized in Figure 2.

## 4. Functions of BLHs in the Response to the Environment

### 4.1. BLHs Involved in Light Responses

Light is an important regulatory signal during plant development. There are a large number of important regulatory factors involved in light signal transduction [104,105]. These regulatory factors cooperate with photoreceptor proteins, such as red and far-red sensing phytochromes, to participate in the growth and development of plants [106].

**Figure 3:**
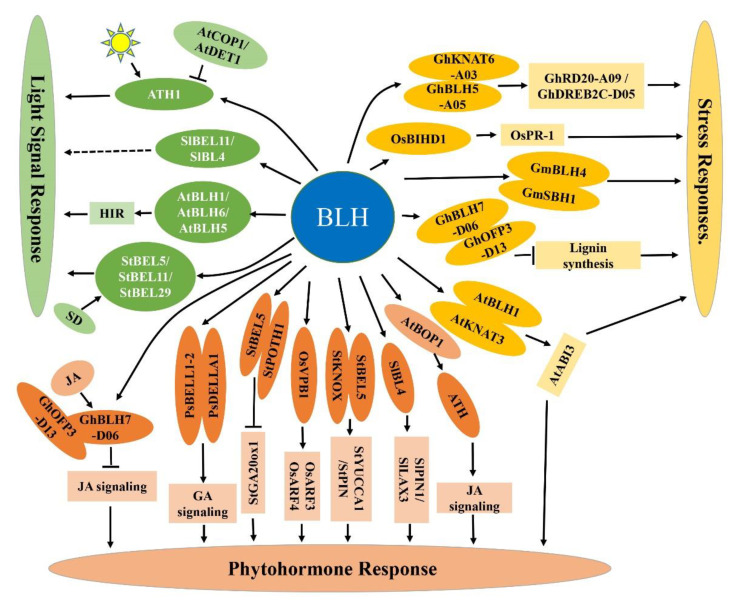
Role of BLHs in light, stress and hormone signaling pathways. The composition of BLH signal pathway is divided into three parts, which is represented by three different colors. Light signals are described in green, stress signals in yellow and hormone signals in orange. The main downstream genes and downstream signaling pathways are described in rectangular boxes. BLH protein and its interacting proteins were described by oval box. Arrows indicate promotion and vertical lines indicate repression. Gene prefixes represent different species (At: *Arabidopsis thaliana*; Sl: *Solanum lycopersicum*; St: *Solanum tuberosum*; Gh: *Gossypium hirsutum*; Ps: *Medicago truncatula*; Os: *Oryza sativa*; Gm: *Glycine max* L.).

Studies have shown that *ATH1* plays an important role in the regulation of light-induced gene expression and photomorphogenesis in *Arabidopsis thaliana* [107,108,109,110]. The expression of *ATH1* is positively regulated by light. In the dark-grown *cop1 det1* double mutants, the expression level of *ATH1* increased, indicating that the expression of *ATH1* is negatively regulated by COP1 (constitutive photomorphogenesis 1) and DET1 (de-etiolated 1). Genetic analysis shows that ATH1 may be an important downstream component of COP1 and DET1 signal transduction pathway [109] (Figure 3). Phytochrome A-mediated plant response has two stages, namely, the very low flux response (VLFR) and the high irradiation response (HIR). BLH1 can specifically regulate the HIR of phytochrome A. In addition, *BLH5*, a homolog of *BLH1*, showed the strongest similarity with *BLH1* and *blh5* mutation also reduces the HIR [111]. In potato, the movement of *StBEL5* mRNA to stolons is induced by short-day photoperiods [43]. The promoter of *StBEL5* contains many light response elements, which provides evidence for *StBEL5* to play a role in photoperiod regulation. Studies have shown that the transcriptional activity of StBEL5 in leaves is not only induced by white light, but also induced by red and blue light, while the far-red light has no effect to *StBEL5* expression [112]. The movement of *StBEL11* and *StBEL29* mRNAs was induced by short-day conditions [45]. The silencing of *SlBEL11* or *SlBL4* in tomato changes the expression level of many photosynthetic-related genes. This result shows that *SlBEL11* and *SlBL4* are also involved in the light signal response [40,41].

### 4.2. BLHs Involved in Plant Resistance to Stress

Plant adaptation to environmental stress mainly depends on the complex molecular regulatory network, including stress signal perception and transmission, stress-response-related gene expression and metabolite synthesis [106]. Studying the molecular mechanism of plant signal transmission under stress is of great significance for breeding stress-resistant crops [113]. *BLH* genes plays an important regulatory role in the plant abiotic stress response. In Arabidopsis, *BLH1* is involved in salt stress response. During seed germination and early seedling development, the sensitivity of the *blh1* mutant to salt decreased, while the sensitivity of a *BLH1* overexpressing line to salt increased [114]. Study has shown that *BLH8* is involved in the abiotic stress response. The *blh8* mutant specifically showed a leaf chlorosis phenotype under ion stress (especially Na^+^ and K^+^), but the plant roots are unaffected [115]. Overexpression of rice *OsBIHD1* in tobacco significantly increased the sensitivity of transgenic plants to salt and oxidative stress, showing the cell membrane damage phenotype under oxidative stress [46]. Apple *MdBLH4.1*, *MdBLH9.1*, *MdBLH8.1*, *MdBLH8.3* and *MdBLH11.1* and poplar *PtTALE5* (homologous gene of Arabidopsis *BLH7*) showed significant changes in gene expression under different concentrations of NaCl and *MdATH1.1,* and *MdBLH7.2* showed a significant decrease in expression under mannitol treatment [59,116]. Cotton *GhBLH5-A05* is a positive regulator of drought stress. Overexpressing *GhBLH5-A05* in Arabidopsis and cotton resulted in stronger drought tolerance. Study has also showed that GhBLH5-A05 could interact with GhKNAT6-A03 to promote the expression of drought stress response genes *GhRD20-A09* and *GhDREB2C-D05*, so as to enhance the tolerance of cotton to drought stress [54] (Figure 3). When soybean *GmBLH4* was overexpressed in Arabidopsis under HTH (high temperature and high humidity), the stress, seed vigor and germination rate of transgenic lines were significantly higher than those of wild type. The KNOX protein GmSBH1 plays an important regulatory role in soybean response to HTH stress environments. The study also shows that GmBLH4 interacts with GmSBH1, resulting in the formation of a complex to jointly regulate the response of soybean to HTH stress [56].

*BLH* genes are also involved in the response of plants to biological stress. A large number of studies have shown that the accumulation of lignin and plant hormone signals are directly related to the resistance of plants to *Verticillium* [117]. The cotton GhBLH7-D06 negatively regulates the resistance of cotton to *Verticillium*. Silencing *GhBLH7-D06* by VIGS (virus-induced gene silencing) induced the expression of lignin synthesis and hormone-signal-related genes, which enhanced the resistance of cotton to *Verticillium* [53]. The promoter of the *StBEL5* gene contains trauma response elements, such as the W, WUN and G-boxes, indicating that the expression of *StBEL5* may be induced by trauma. Chatterjee et al. analyzed the GUS activity of *StBEL5* transgenic plants and found that the transcription of *StBEL5* was activated by trauma and pests [112]. Rice *OsBIHD1* plays an important role in the resistance response of *Magnaporthe grisea*, and the expression of *OsBIHD1* in rice seedlings increased significantly after inoculation with *Magnaporthe grisea* [48]. Overexpression of *OsBIHD1* in tobacco resulted in activation of the expression of defense-related genes *PR-1*, which enhanced the disease resistance of rice to viruses and the oomycete pathogen [46] (Figure 3).

## 5. BLHs Involved in Phytohormones Biosynthesis and Signaling

Plant hormones are an important part of the complex molecular network regulating plant growth and development, and transcriptional regulation plays an important role in plant hormone-mediated signal transduction pathways [118]. BLH transcription factors have been shown to participate in plant hormone-mediated growth and development, especially abscisic acid (ABA), indole-3-acetic acid (IAA), gibberellin acid (GA) and jasmonic acid (JA).

### 5.1. BLHs Involved in ABA Response

In Arabidopsis, *BLH1* is involved in ABA-mediated seed germination and seedling development. Overexpression of *BLH1* promotes the expression of ABA response genes *ABI3* (*abscisic acid insensitive 3*) and *ABI5* in transgenic plants. The complex formed by the interaction between BLH1 and KNAT3 can activate the expression of *ABI3,* to enhance the ABA response in plants and jointly regulate seed germination and seedling growth [114,119].

### 5.2. BLHs’ Regulation of IAA Response

BP is a potential interaction partner of BLH6, and their expression regions overlap partially in roots. The overexpression of *BP* affects the redistribution of auxin in plants, resulting in the abnormal growth of plant lateral roots. These results show that BLH6 and BP are involved in the plant hormone-dependent root regulation network [26]. The BEL1 transcription factor plays an important role in the ovule hormone network, especially the auxin and cytokinin signaling pathways. In addition, PIN1 is an auxin efflux-promoting factor [120], and the ectopic expression of *PIN1* caused the abnormal ovule development in the *bel1* mutant [121]. The auxin response factor *ETT* (*ETTIN/ARF3*) is important for the establishment of pistil apical polarity, which ensures the normal synthesis and distribution of auxin in plant organs [122,123]. *SPT* (*SPATULA*) and *IND* (*INDEHISCENT*) are important *bHLH* family regulators of pistil development [124,125,126]. Recent research results show that ETT, IND, BP, RPL and SEU jointly regulate the distribution of auxin in different organs [85]. In rice, *VPB1* regulates the arrangement of panicles and branches, and the distribution or content of auxin in the *vpb1* mutant changed. This resulted in decreased activity of the inflorescence meristem, and finally led to the disorder of initiation and arrangement of branch meristems. Transcriptome analysis showed that *VPB1* affected the expression of hormone signaling pathway genes, such as *ARF3*, *ARF4* and *ARF12*. This result showed that *VPB1* plays a role in plants, mainly by regulating meristem development-related genes, to affect the activity of inflorescence primordium and subsequent differentiation [28].

The phloem transport of *StBEL5* mRNA is related to auxin synthesis and signal transduction. StBEL5 cooperates with KNOX to induce the expression of auxin biosynthetic gene *YUCCA1* and transport gene *PIN* [43] (Figure 3). Silencing tomato *SlBL4* enhanced the sensitivity of plants to IAA, and exogenous IAA hormone treatment inhibited the early abscission phenotype of flower stems of *SlBL4* RNAi plants. Transcriptome analysis shows that SlBL4 regulates the expression of many genes related to auxin signal transduction, and experimental analysis shows that SlBL4 can activate the transcription of auxin transport-related genes *PIN1* and *LAX3* [42] (Figure 3).

### 5.3. BLHs’ Regulation of GA Response

DELLA protein plays an important role in the gibberellin regulatory network. Research has found that PsBELL1–2 interacts with PsDELLA1 to regulate the nodulation process in *Pisum sativum*. The study also showed that PsBELL1–2 could interact with PsKNOX9, and both may be regulated by NIN, which is one of the most important regulatory factors of nodule organogenesis and infection [60]. In potato, StBEL5 interacts with POTH1 (potato homeobox 1) to regulate potato development by regulating GA and cytokinin levels. Ga20ox1 encodes a key enzyme in the gibberellin biosynthesis pathway. Overexpression of *StBEL5* in potato reduces the *GA20ox1* mRNA level at the stolon tip and increases the cytokinin level in the shoot [15]. The StBEL5-POTH1 protein complex can bind to the promoter of *GA20ox1* and negatively regulate its expression [124] (Figure 3). In cotton, *GhBEL1*, *GhBLH1* and *GhBLH6* also participate in the gibberellin signal regulation network, but the specific mechanism is not clear [52].

### 5.4. BLHs’ Regulation of JA Response

The disease resistance response of plants involves a complex signal transduction network, regulated by a series of signal molecules. Cotton *GhBLH7-D06* negatively regulates cotton resistance to *Verticillium* and jasmonic acid (JA) and can induce the expression of *GhBLH7-D06*. Silencing *GhBLH7-D06* can significantly increase the expression level of genes related to JA biosynthesis and signal transduction genes, such as *GhLOX1-A08*, *GhLOX2-A05* and *GhLOX3-A09*, and enhance the resistance of cotton to *Verticillium* [53]. BLADE-ON-PETIOLE1 (BOP1) is a lateral organ boundary protein and it can directly activate ATH1 under the action of cofactors, to increase the content of JA in plants by promoting the expression of JA biosynthetic genes [69] (Figure 3).

The essential role played by BLH proteins in many aspects of plant growth and development, environmental and plant hormone signaling, is summarized in Figure 3. At present, research on BLHs and their interacting networks is incomplete and needs further exploration.

## 6. Conclusions and Prospects

### 6.1. BLHs Are Important Candidate Genes for Molecular Breeding

The BLH gene family has a variety of functions in plant development and stress response. Many *BLHs* can be used as high-quality candidate genes for molecular breeding. For example, *ZmBLH12* and *ZmBLH14* play an important regulatory role in maintaining the development of axillary meristem in maize. Proper expression regulation of *ZmBLH12* and *ZmBLH14* may be helpful to cultivate maize of ideal plant type [51]. In potatoes, *StBEL5* is a candidate gene for cultivating potato varieties with a short growth cycle, which is of great significance to alleviate the world’s food problems [15]. In addition, overexpression of *SlBL4* improves the firmness of tomatoes, which helps to reduce the loss caused by tomato transportation [41]. *Populus pilosa BLH* family genes *PtrWBLH1/2* and *Populus euphratica PeuBELL15* are important regulators in the expression of plant primary and secondary cell wall-related genes [64,99]. Through the regulation and expression of these genes, we can improve and select improved wood varieties, with rapid growth and high wood biomass, so as to promote the high-quality and efficient utilization of wood. In *Medicago truncatula*, gene editing of *PINNA1* may be helpful for cultivating forage varieties with higher leaf yield, which has important economic value [61]. In addition to the regulation of plant growth and development, *BLHs* can also be used as candidate genes in the cultivation of plant varieties resistant to biotic and abiotic stress. Screening varieties with high expression of *GhBLH5-A05* and low expression of *GhBLH7-D06* and *GhBLH6* in cotton may hasten the production of varieties with drought resistance and *Verticillium* resistance [53]. In addition, due to the specificity of promoter expression, some *BLHs* are expressed in specific locations, such as *ATH1*, *PNY* and *OsBLH6* [13,49,71]. These promoters can be utilized to direct transgenes expression, in the right place, at the right time, to precisely regulate the relevant phenotype, maximize the positive influence and minimize any adverse effects of the gene.

### 6.2. The Post-Translational Modification of BLHs Remains to Be Further Studied

In addition to transcriptional regulation, post-translational modifications play an important role in the regulatory network of transcription factors. Recent studies have shown that an E3 ubiquitin ligase (MdPUB24) in apple promotes the degradation of MdBEL7 and regulates the greening process of the apple fruit [57]. However, there are few studies on the post-translational modification of BLHs. How a large number of BLHs with different functions in plants are modified has not been reported. Whether BLHs are regulated by E3 ubiquitin ligases other than PUB needs to be further explored. Whether there are other protein post-translational modifications in BLHs also needs further research. With the continuous advancement of scientific research technology, combining different developmental stages or stress conditions of plants through proteomics methods will help us to identify potential modified proteins of BLHs more quickly. Understanding the post-translational modification process of BLHs will help us better study the functions of the BLH gene family and lay the foundation for the construction of the complex regulatory networks of BLHs.

### 6.3. The BLHs Regulatory Network Requires Further Study

BLHs are an important class of TFs. To date, only a few target genes regulated by BLHs have been analyzed. For example, BLH1 in Arabidopsis directly targets the *AtABI3* promoter and increases its expression to regulate seed germination [114]. SlBEL4 and SlBEL11 directly inhibit the expression of genes related to chloroplast development and negatively regulate chloroplast development in tomato fruits [40,41]. OsVPB1 in rice regulates the inflorescence structure of rice by directly inhibiting the expression of *OsBOP1* [28]. However, there are still many downstream genes directly regulated by BLHs that have not been identified or reported. These target genes of BLHs need to be studied in much greater detail. In addition, the regulatory principle and mechanism of BLHs in plant tissues require further clarification. Our understanding of the regulatory pathways is not deep enough, and the regulation at different levels and interactions between proteins are unclear. The identification of BLHs’ interacting proteins is essential to explain how these genes affect the development of different organs. In the future, it will be necessary to combine genomics, transcriptomics and molecular genetics to study the function of BLHs in crop growth and development. Bioinformatics and system analysis in different species are of great significance for the discovery of new BLHs and function prediction. With the advancement of high-throughput sequencing technology and genomics research, a large number of target genes of BLHs are expected to be identified and the regulatory network of the BLHs are also expected to be clarified in detail. This will be of great significance for future crop genetics and breeding.

## Figures and Tables

**Table 1:** Identification and characterization of BLH genes in different plant species.

Species	Gene Number	Characterized to Date	Function	Reference
*Physcomitrium patens*	4	*PpBELL1*	Sporophyte development	[20,21,22,23]
*Selaginella moellendorffii*	2	/	Unknown	[21]
*Gnetum gnemon*	4	/	Unknown	[24]
*Vandenboschia speciosa*	11	*VsBELL4*, *VsBELL6*, *VsBELL10*	Gametophytic and the sporophytic developmental	[25]
*Arabidopsis thaliana*	13	*BEL1*, *ATH1*, *BLH1*, *BLH2*/*SAW1*, *BLH3*, *BLH4*/*SAW2*, *BLH6*, *BLH8*/*PNF*, *BLH9*/ *PNY*/*BLR*/*RPL*/*VAN*/*LSN*	Meristems and inflorescence development; plant morphogenesis; cell wall; ovules; embryo; light signal; abiotic stress and hormone signaling	[6,7,11,13,26,27,28,29,30,31,32,33,34,35,36,37,38,39]
*Solanum lycopersicum*	14	*SlBL4*, *SlBEL11*	Fruit chlorophyll; cell wall and hormone signaling	[40,41,42]
*Solanum tuberosum*	13	*StBEL5*, *StBEL11*, *StBEL29*	Phloem-mobile mRNA signals; yield; photoperiod and hormone signaling	[15,16,43,44,45]
*Daucus carota* L.	14	/	Unknown	[20]
*Oryza sativa* L.	14	*OsSH5*/*VBP1*/*RI*, *OsBIHD1*, *OsRIL1*, *qSH1*, *OsBLH6*	Seed shattering; regulation of inflorescence architecture and meristem maintenance; Secondary cell wall biosynthesis; morphological development; stress response	[11,21,28,46,47,48,49,50]
*Zea mays* L.	18	*ZmBEL12*, *ZmBEL14*	Meristem maintenance; leaf morphology;	[51]
*Gossypium* *hirsutum* L.	50	*GhBLH5-A05*, *GhBLH6-A13* *GhBLH7-D06*	Secondary cell wall biosynthesis; morphological development; drought stress; virus response and hormone signaling	[52,53,54]
*Glycine max* L.	34	*GmBLH4*	Morphological development; stress response; nodule development	[55,56]
*Malus domestica* L.	19	*MdMDH1*/*MdBLH4.1*, *MdBLH7*, *MdBLH9.1*, *MdBLH8.1*, *MdBLH8.3*, *MdBLH11.1*, *MdATH1.1*, *MdBLH7.2*	plant morphogenesis; chlorophyll degradation; salt response; drought stress	[57,58,59]
*Medicago truncatula*	14	*PINNA1*, *MtBELL1–2*, *MtBELL1–3*, *MtBELL1–4*	leaf morphology; nodulation	[60,61]
*Pisum sativum* L.	14	*PsBELL1–2*, *PsBELL1–3*, *PsBELL1–4*	nodulation; hormone signaling	[60]
*Brassica rapa* L.	14	/	Unknown	[62]
*Camellia chekiangoleosa*	12	*CchBLH6*	Fruit lignification	[63]
*Populus trichocarpa*	20	*PtrWBLH1*, *PtrWBLH2*	Salt stress	[64]
*Juglans regia* L.	17	*JrTALE3*, *JrTALE5*, *JrTALE7*, *JrTALE10*, *JrTALE15*, *JrTALE22*	Flower bud development	[65]
*Punica granatum* L.	9	*PgTALE8*, *PgTALE14*	Ovule development; inflorescence development;	[66]
*Cicer arietinum* L.	12	/	Unknown	[55]
*Lotus japonicus*	6	/	Unknown	[55]
